# Studying the Effects of Reproductive Hormones and Bacterial Vaginosis on the Glycome of Lavage Samples from the Cervicovaginal Cavity

**DOI:** 10.1371/journal.pone.0127021

**Published:** 2015-05-20

**Authors:** Linlin Wang, Sujeethraj Koppolu, Catherine Chappell, Bernard J. Moncla, Sharon L. Hillier, Lara K. Mahal

**Affiliations:** 1 Biomedical Chemistry Institute, Department of Chemistry, New York University, New York, NY, 10003, United States of America; 2 Department of Obstetrics, Gynecology and Reproductive Sciences, Magee-Womens Hospital of the University of Pittsburgh Medical Center, Pittsburgh, PA, 15213, United States of America; 3 Magee-Womens Research Institute, Pittsburgh, PA, 15213, United States of America; Indian Institute of Science, INDIA

## Abstract

The cervicovaginal fluid (CVF) coating the vaginal epithelium is an important immunological mediator, providing a barrier to infection. Glycosylation of CVF proteins, such as mucins, IgG and S-IgA, plays a critical role in their immunological functions. Although multiple factors, such as hormones and microflora, may influence glycosylation of the CVF, few studies have examined their impact on this important immunological fluid. Herein we analyzed the glycosylation of cervicovaginal lavage (CVL) samples collected from 165 women under different hormonal conditions including: (1) no contraceptive, post-menopausal, (2) no contraceptive, days 1-14 of the menstrual cycle, (3) no contraceptive, days 15-28 of the menstrual cycle, (4) combined-oral contraceptive pills for at least 6 months, (5) depo-medroxyprogesterone acetate (Depo-Provera) injections for at least 6 months, (6) levonorgestrel IUD for at least 1 month. Glycomic profiling was obtained using our lectin microarray system, a rapid method to analyze carbohydrate composition. Although some small effects were observed due to hormone levels, the major influence on the glycome was the presence of an altered bacterial cohort due to bacterial vaginosis (BV). Compared to normal women, samples from women with BV contained lower levels of sialic acid and high-mannose glycans in their CVL. The change in high mannose levels was unexpected and may be related to the increased risk of HIV-infection observed in women with BV, as high mannose receptors are a viral entry pathway. Changes in the glycome were also observed with hormonal contraceptive use, in a contraceptive-dependent manner. Overall, microflora had a greater impact on the glycome than hormonal levels, and both of these effects should be more closely examined in future studies given the importance of glycans in the innate immune system.

## Introduction

The mucosal lining of the female genital tract provides a robust barrier to infection from pathogens such as HIV-1 [1, 2]. Cervical mucus, a natural hydrogel consisting predominantly of water (95%-98%) and large and structurally complex mucin glycoproteins (2%-5%), is secreted into the vagina providing lubrication and a natural barrier to microorganisms and viruses [3–6]. Cervicovaginal fluid (CVF) contains this mucus along with an assortment of other anti-microbial glycoproteins including S-IgA, IgG, cathepsin G, lysozyme and lactoferrin [7–11]. Glycosylation of proteins in the CVF influence their stability, activity and function [12]. For example, mannose structures on S-IgA in vaginal fluid act as an alternative ligand for uropathogenic type-1 *Escherichia Coli* inhibiting vaginal colonization and subsequent urinary tract infection[13]. Thus, glycosylation plays an important role in the anti-microbial properties of the CVF.

Multiple factors may influence the glycomic composition of the CVF including hormones and vaginal microflora. Oral contraceptives have been shown to regulate the glycosylation of serum glycoproteins such as α1-acid glycoprotein [14]. In one of the only studies on the CVF glycome, changes in sialylation were observed in cervical mucin O-glycans at ovulation. However, few differences were observed at other time points [3]. Microflora may also play a role in CVF glycome composition. Women with bacterial vaginosis (BV), in which the balance between *Lactobacillus* species and competing anaerobic bacteria shifts towards the anaerobes [15], display high levels of vaginal sialidase, first reported by *Briselden et al*. [16, 17]. These enzymes cleave the negatively charged sugar sialic acid from terminal glycans of glycoproteins in the CVF, changing the glycan composition of the CVF and increasing proteolysis of innate immune factors such as S-IgA and lactoferrin [18].

To date, no systematic study has examined the effects of exogenous hormones and microflora on the vaginal glycome. Herein, we utilize lectin microarrays [19, 20], our high-throughput glycomic analysis platform, to profile the glycosylation patterns of cervicovaginal lavage (CVL) samples from 169 women. The sample cohort includes women on different hormonal contraceptives and with different microflora. Lectin microarrays, in which carbohydrate binding proteins are arrayed, are a versatile glycomic platform that has been used to analyze the glycosylation of samples from bacteria to human cancer tissues [21–26]. Our lectin microarray data demonstrates that while both exogenous hormones and microflora affect the glycome, the strong impact of microflora on the glycome, confounds assessment of hormonal effects. Decreased high mannose levels were observed in the CVL of women with BV, which may increase susceptibility to other pathogens. This study sets the stage for more detailed analysis of the effects of both hormone and individual microbes on the glycome of vaginal fluids and its function in innate immunity against pathogens.

## Materials and Methods

### Study Population

Following Institutional Review Board approval by the University of Pittsburgh (#PRO11020218), written informed consent was obtained from subjects enrolled in our study. Women were excluded if: they were breastfeeding or pregnant; presented vaginal symptoms; had a hysterectomy; had been diagnosed with any cervical or vaginal infections or had used any antimicrobials in the past 14 days; had used any vaginal devices or vaginally-applied products (excluding tampons) in the past week. Upon enrollment the women had: an OraQuick advance rapid HIV test; a pregnancy test; their demographic information was recorded; height and weight taken and medical, gynecologic and sexual histories taken. Cervicovaginal lavage (CVL) was collected from 169 women. 4 of 169 samples were excluded.

These women were characterized into six groups: (1) post-menopausal; (2) not contracepting on days 1–14 of the menstrual cycle; (3) not contracepting on days 15–28 of the menstrual cycle; (4) combined oral contraceptive; (5) DMPA (medroxyprogesterone acetate, Depoprovera); and (6) Mirena IUD (levonogerestrol Intrauterine Device) usage. These data were also stratified according to microflora status using the Nugent score [27].

### Sample Collection

First a vaginal swab was taken for a Gram Stain evaluation for microflora status using the Nugent method of scoring [27]. Cervicovaginal lavage specimens (CVL) were collected in 10 mL of normal saline (Hospira, Inc. Lake Forest, IL). The saline and a syringe were used to gently wash the cervix and vaginal vault. The CVL was collected and placed in a 15 mL plastic centrifuge tube and stored at 4°C until processing. Within one hour the samples were transported to the laboratory. Upon receipt in the laboratory, CVLs were dispensed into 2 mL cryovials. All samples were stored at -80°C.

### Sample labeling

The CVL samples (100 μg protein based on Lowry) were labeled with NHS-Cy5 dye (6 μg GE Healthcare Life Sciences, Piscataway, NJ) in 100 mM NaHCO_3_ buffer, pH 9.3. A pooled sample of the CVL was labeled with NHS-Cy3 dye (60 μg per mg of protein) for use as the reference. Samples were incubated for 45 min at room temperature with gentle shaking to allow labeling and the free dye was then quenched by addition of 2 M Tris buffer, pH 6.8 (final concentration 250 mM). Samples were then dialyzed overnight against PBS at 4°C using a Microdialyzer (3,000 MW membrane, Spectrum Laboratories). All the steps were performed in the dark. Protein concentrations of samples were obtained after dialysis using the microBCA assay (Pierce).

### Lectin Microarray Analysis

#### Print conditions

Lectin microarrays were printed as previously described [20]. In brief, 92 lectins and antibodies (S1 Table) were kept on ice during preparation and transferred to 384-well microplates for printing. Samples were maintained at 12°C and printed on Nexterion H slides (SCHOTT North America, Elmsford, NY) using a Nanoplotter 2.1 piezoelectric printer (GeSIM, Germany) with a pico-tip (Quantum analytics, Foster City, CA) at 45–50% humidity. Three spots per protein were printed in each of the 24 arrays per slide. After printing, slides were incubated at RT for 2 hr to obtain maximum coupling to the NHS ester surface. The lectins and antibodies, print concentrations and buffers are listed in S1 Table. Lectin microarrays were tested for quality using Cy5-labeled glycoprotein standards (10 μg asialofetuin, fetuin and RNase B).

#### Microarray hybridization

Printed slides were blocked with 25 mM ethanolamine in 100 mM sodium borate (pH 8.5) for 1 hr at RT in a coplin jar with gentle shaking. Slides were washed 3 × with PBST (PBS with 0.005% Tween 20) and 1 × with PBS for 5 min each and then dried using a slide spinner (Labnet International). An ArrayIt multi-well hybridization cassette (Arrayit Corporation, Sunnyvale, CA) was used to create 24 distinct arrays for hybridization. 2.5 μg of Cy5-labeled CVL sample was incubated with equal amounts of Cy3-labeled reference in 100 μl total volume PBST. Samples were added to each array and incubated for 2 h at RT with gentle agitation. Sample solutions were then aspirated and slide chambers were washed 5 × with PBST (100 μL, 3 min, RT), followed by removal of the hybridization chamber and a final wash with PBS (5 min in a coplin jar). The slide was spun-dry and scanned in the Cy3 (ex/em 532/550–600 nm) and Cy5 (635/655–695 nm) channels with a Genepix 4300A slide scanner (Molecular Devices, Sunnyvale, CA). Data were extracted using Genepix 7 (Molecular Devices) and processed with Microsoft Excel 2011.

#### Microarray data analysis

The background-subtracted median fluorescence of the three replicate spots per protein was tested for outliers using the Grubbs outlier test with α = 0.05. The average value was determined for the three replicates after excluding the outliers if there were any. The log_2_ values of the average signals were median-centered over the array in each channel to account for differences in labeling efficiency [28]. The values obtained were log_2_ ratios for each lectin or antibody. Hierarchical clustering of the processed data (S1 Data) was performed using Pearson correlation coefficient with average linkage analysis by Cluster 3.0 [29] and the data sets were visualized with Java Treeview [30]. We excluded from the analysis probes whose average signal was not higher than the median of the array for at least of 10% of samples as these were considered inactive. This resulted in the exclusion of 22 lectins from further analysis. Lilliefors test [31–33] was used to test the normality for sample distributions. Data was considered normal by this standard. P values were calculated with one-way ANOVA [34]. All the box plots were made with Matlab 2013a.

## Results and Discussions

### Study Design

Cervicovaginal lavage (CVL) samples, which represent a comprehensive collection of fluid and mucus from the lower reproductive tract [35], were collected from 169 women under different hormonal and reproductive conditions including: (1) post-menopausal, 29 women; (2) days 1–14 of the menstrual cycle, 27 women; (3) days 15–28 of the menstrual cycle, 26 women; (4) combined oral contraceptive for at least 6 months, 27 women; (5) depo-medroxyprogesterone acetate (Depo-Provera) injections for at least 6 months, 28 women; (6) levonorgestrel Intrauterine Device (IUD) usage for at least 1 month, 28 women [36]. Women in groups 1–3 were free of hormonal contraceptives. Four of the 169 samples were excluded from further analysis due to failure to meet the inclusion criterion. One sample was excluded due to *Chlamydia trachomatis*. The other three samples were excluded because three women participated in the sample collection protocol for two different periods of the same menstrual cycle, i.e. their samples were collected for groups 2 and 3. The vaginal microflora of study participants was evaluated using the Nugent scoring system to determine bacterial vaginosis (BV) status [27]. Normal microflora was defined as a Nugent score of 0–3, intermediate: 4–6 and BV: 7–10. In a recent study, the Nugent score was found to accurately reflect shifts in the microflora composition of the vagina [15]. The Nugent score is validated only for use among women of reproductive age, so it does not apply to postmenopausal women [27]. Consequently postmenopausal women were excluded from our comparative analysis of Normal and BV samples.

### Lectin Microarray Analysis of the CVL

**Fig 1:**
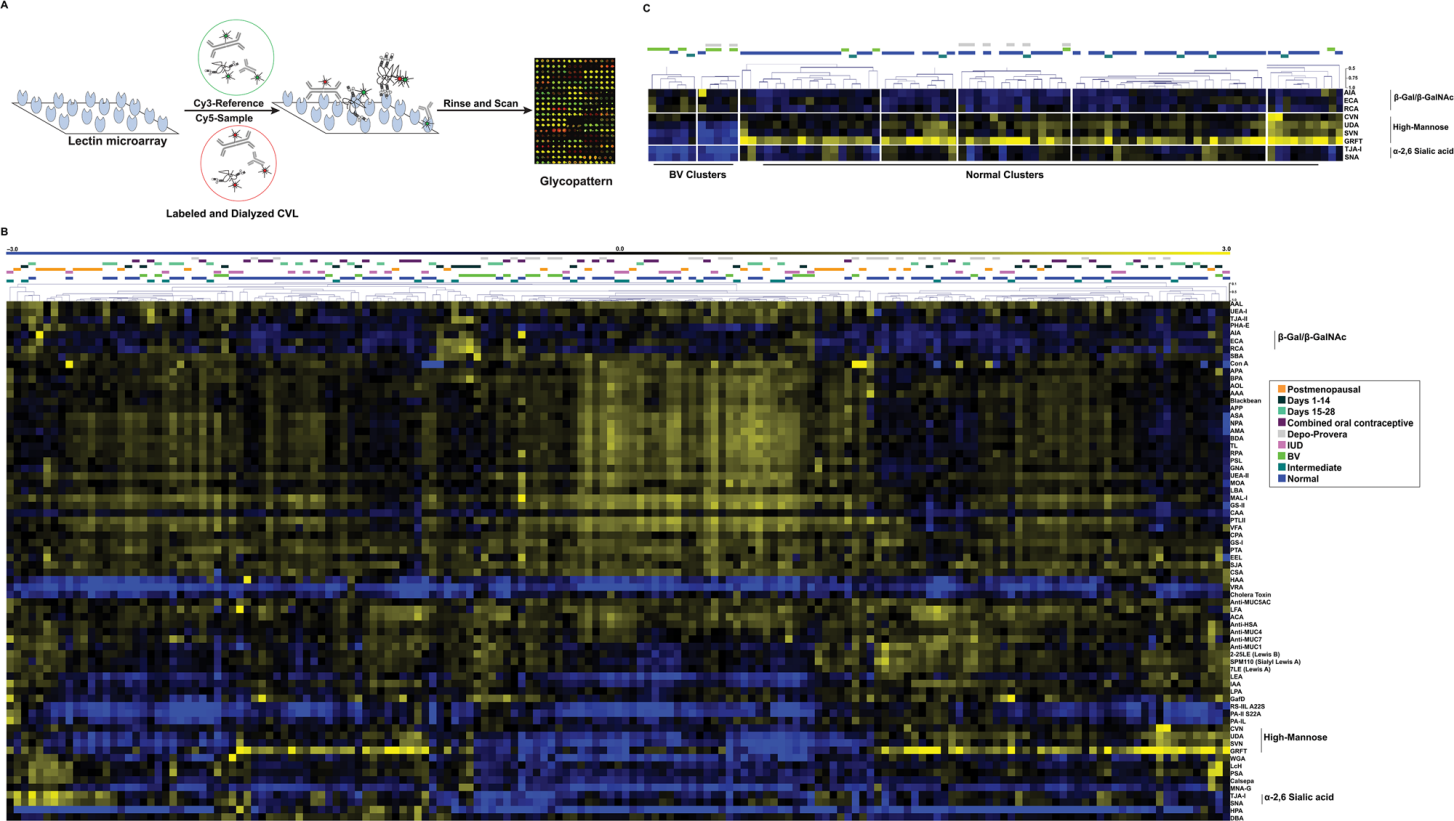
Lectin Microarray Analysis of CVL. (A) Experimental scheme. Equal amounts of Cy5-CVL sample and Cy3-pooled CVL reference were analyzed on the lectin microarray. (B) Heat map of hierarchical clustering. Median-normalized log_2_ ratios (Sample (S) /Reference (R)) of 165 CVL samples were hierarchically clustered using Pearson correlation coefficient as the distance metric and average linkage analysis (yellow, log_2_(S) > log_2_(R); blue, log_2_(R) > log_2_(S)). Samples are color coded by hormonal and microbial status across the top: Postmenopausal (orange), Days 1–14 (black), Days 15–28 (light teal), Oral contraceptive (purple), Depo-Provera (gray), IUD (pink), BV (green), Intermediate (dark teal), Normal (blue). (C) Select sample and lectin clusters showing BV vs. Normal patterns. Pearson correlation coefficient scale is shown.

In lectin microarray analysis, we label the glycoproteins in our samples with Cy5-dye through lysine coupling to the corresponding NHS-ester. The labeled glycoproteins are then mixed with a Cy3-labeled biological reference and incubated with our microarrays to obtain a semiquantitative dual-color analysis that is accurate for relative glycomic composition (Fig 1A) [37–39]. Cervical vaginal lavage fluid is known to contain large amounts of free sugars, mainly glucose, mannose and glucosamine [7]. As high concentrations of these monosaccharides can inhibit lectins, such as those on our microarrays, we tested whether dialysis of labeled CVL samples would alter the resulting microarray profiles. In initial studies, we observed lower signals for some lectins in undialyzed samples presumably due to free sugar content, thus we dialyzed all CVL samples post-labeling using a low molecular weigh cutoff (3 kD, S1 Fig). For our analysis, we combined 2.5 μg of Cy5-labeled CVL sample with equal amounts of a Cy3-labeled pooled reference created from all samples and analyzed them on our lectin microarrays. Median-centered log_2_ (sample/reference) ratios were calculated after data extraction. Normalized data was then hierarchically clustered using the Pearson correlation coefficient as the distance metric and average linkage analysis to generate the heatmap shown in Fig 1B.

Initial examination of the heatmap suggested that both microbial status and hormonal levels impact the glycome. Samples most clearly segregated by microbial status (Fig 1C). This pattern was established by three sets of lectins: β-galactose (Gal)/ β-*N*-acetyl-D-galactosamine (GalNAc) (AIA, ECA, RCA, a list of lectin abbreviations and rough specificities is shown in S1 Table), high mannose (CVN, UDA, SVN, GRFT) and α2, 6-sialic acid binders (TJA-I and SNA). In contrast, a clear pattern was difficult to discern for hormonal regulation, although some clustering was observed for postmenopausal women (PM) and women on Depo-Provera (Depo). Microbial status interfered with our assessment of the role that hormones play in establishing the CVL glycome. We observed two distinct Depo clusters (one with BV and one without) with different glycan profiles in our large dataset (Fig 1C), arguing that microbial status may predominate over hormones in the control of glycosylation. We cannot address the microbial status of the postmenopausal women in this study, so conclusions on the role of hormone and/or microbial status on glycosylation in that cohort are not possible, thus we excluded these samples from further analysis. Based on our findings, we undertook a more detailed statistical analysis of our data to better define the roles of microbial status (BV/normal) and hormones on specific glycosylation signatures in the CVL.

### Bacterial Vaginosis has a Strong Effect on Glycosylation in the CVL

**Fig 2:**
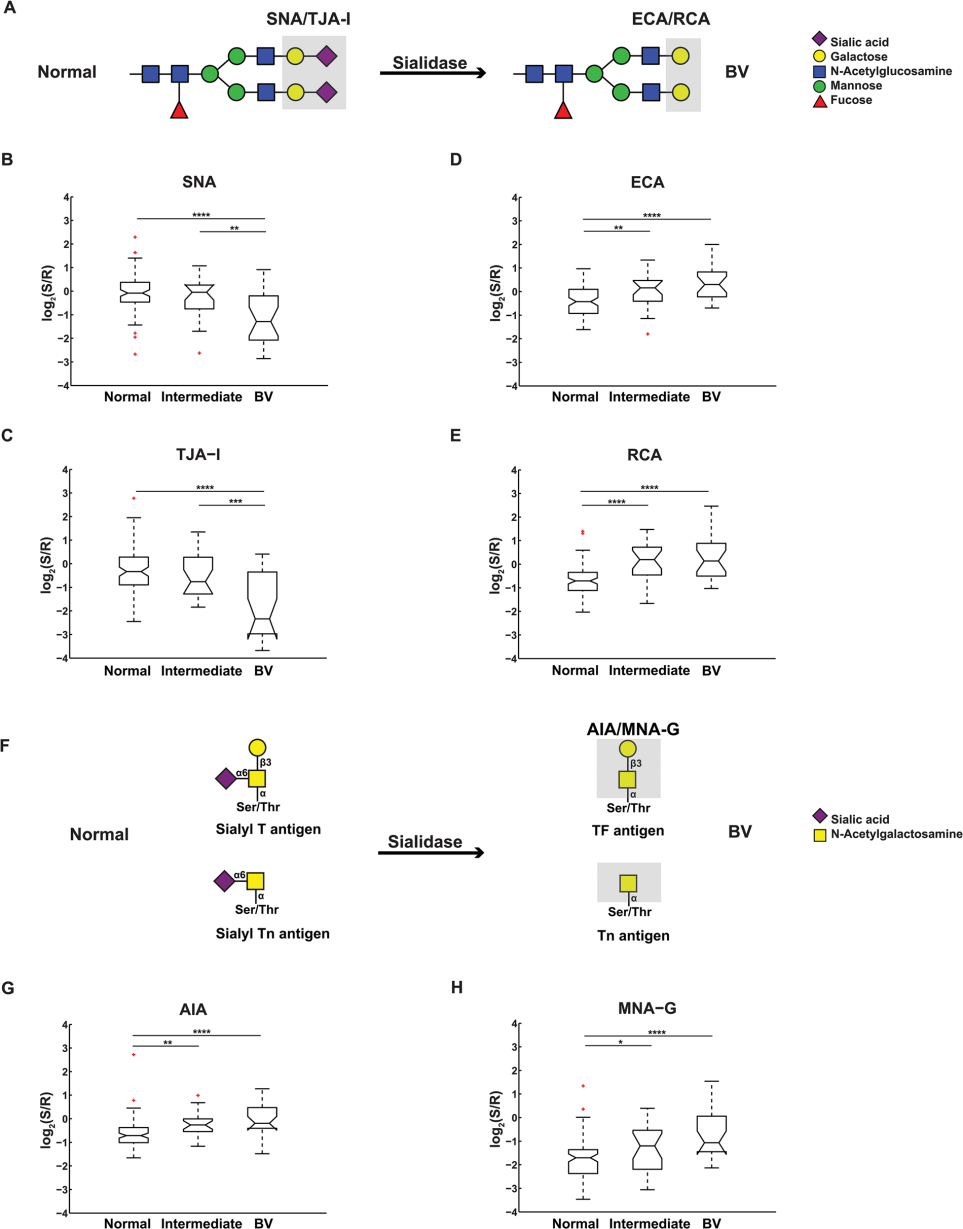
Effects of vaginal microflora on sialylation. (A) Representative *N*-linked glycan before and after sialidase digestion. Lectin binding determinants are shaded in grey with lectin names indicated. (B-E) Notched boxplot representation of binding levels of (B) SNA, (C) TJA-I, (D) ECA, (E) RCA to Normal, Intermediate and BV sample cohorts. (F) Representative *O*-linked glycans before and after sialidase. (G-H) Notched boxplot representation of binding levels of (G) AIA and (H) MNA-G. For all plots, significance levels between groups indicated by lines are as follows: *, 0.01< *p* ≤0.05; **, 0.001< p ≤0.01; ***, 0.0001<p≤0.001; ****, p≤0.0001. Outliers are marked in red.

**Fig 3:**
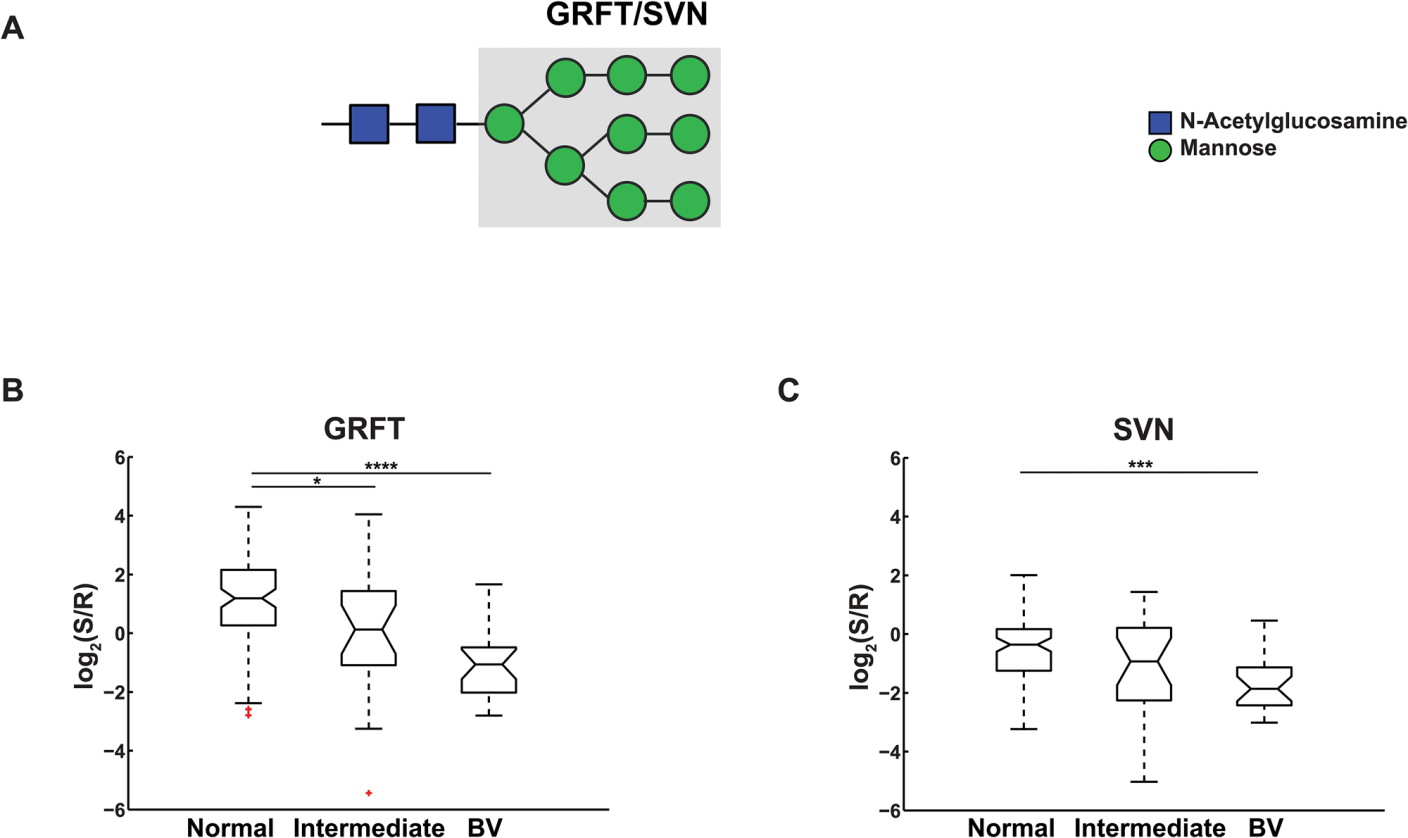
Effects of vaginal microflora on high mannose. (A) High mannose glycan structure. Lectin binding determinants are shaded in grey. (B-C) Notched boxplot representation of binding levels of (B) GRFT and (C) SVN. Significance: *, 0.01< *p* ≤0.05; **, 0.001< p ≤0.01; ***, 0.0001<p≤0.001; ****, p≤0.0001. Outliers are in marked red.

To more closely examine the effects of bacterial vaginosis (BV) on CVL glycosylation in this cohort, we performed ANOVA analysis on a combined cohort of BV (n = 23), intermediate (n = 23) and normal (n = 90) samples, disregarding hormonal status, for each lectin on our microarray. In line with our earlier findings, we observed statistically significant decreases in lectins corresponding to α2,6-sialic acid, and high mannose epitopes and an increase in β-Gal, β-GalNAc binding (Figs 2 and 3). We tested whether hormonal differences in the cohorts could account for these changes as we had a predominance of 2 groups within the BV cohort (days 1–14 of the menstrual cycle and Depo-Provera, n = 8 each, 35% each of cohort). Normal samples from the days 1–14 and Depo-Provera groups did not follow the trends observed in BV (S2 Fig). In addition, comparison of normal vs. BV samples within the each group demonstrated similar effects on the glycome due to aberrant microflora seen in the combined cohort (i.e. decreases in α2,6-sialic acid and high mannose) arguing that the microbiome overrides hormonal effects (S3 and S4 Figs).

Several of these changes are consistent with the known biological effects of BV on the glycome of the vagina. In bacterial vaginosis higher levels of sialidase, an enzyme that cleaves sialic acid molecules from underlying β-Gal and β-GalNAc structures, are observed [6, 16]. In a companion paper, Moncla et al. show higher levels of sialidase activity correlated with BV in these CVL samples. This would cause a loss of sialic acids and an increase in exposed terminal β-Gal and β-GalNAc residues (Fig 2A and 2F). In our data we observed the loss of α2, 6-sialic acid residues (*p* < 0.0001 for both SNA [40] and TJA-I [41], Fig 2B and 2C) and the gain of terminal β-Gal and β-GalNAc structures (β-Gal: ECA [42] and RCA [43], *p* < 0.0001 for both, Fig 2D and 2E; β-GalNAc: AIA [44] and MNA-G [45], *p* < 0.0001 for both, Fig 2G and 2H). We also observed an effect of BV on levels of α2, 3-sialic acid as probed by *Maackia amuerensis* lectin-I (MAL-I) binding but the effect is not statistically significant (*p* = 0.4). Similar results for SNA and *Maackia amuerensis* lectin were observed by enzyme-linked lectin assays (see the accompanying paper by Moncla et al., PONE-D-15-01714). This more mild effect on MAL-I binding may be due to the strong binding of MAL-I to sulfated glycans, which are present in CVL but are not affected by sialidase [3, 46, 47] (S5 Fig). We also observed a gain in binding to terminal β-Gal and β-GalNAc residues, consistent with their exposure by sialidase (Fig 2D, 2E, 2G and 2H). This increase is observed in both the *N*-linked (ECA, RCA) and *O*-linked (AIA, MNA-G) cohorts and is clear even in intermediate samples where the changes in sialic acid are not readily apparent. Levels of α-GalNAc, however, were unaffected by BV (HPA, S6 Fig).

Our data also shows a loss of high mannose residues on glycoproteins of the CVL from women with BV (Fig 3). High-mannose glycans can contain five to nine mannose residues attached to the chitobiose (GlcNAc_2_) core and are early products of *N*-glycan biosynthesis. We observed a significant loss of binding to two algal lectins, *Griffithsin (*GRFT) and *Scytovirin* (SVN), which are both specific to Man_7_-Man_9_ high mannose structures, in the BV cohort (Fig 3 B and C, *p* < 0.0001 and *p* = 0.0002, respectively). This data is supported by work by Moncla et al. (see accompanying paper). Both of these proteins are known anti-viral lectins and are currently being examined for use as microbicides against viruses including HIV-1 and hepatitis-C [48–50]. We do not observe statistically significant differences with other mannose-binding lectins, such as AMA, ASA [51], ConA [52], GNA and NPA [53] (S7 Fig), which can also bind Man_5_-Man_6_, suggesting that this loss is restricted to the Man_7_-Man_9_ subset.

Previous studies have shown that both HIV infection risk and urinary tract infection risk increase with bacterial vaginosis [54–57]. High mannose residues on S-IgA have been proposed to act as a natural inhibitor of urinary tract infections by inhibiting *Escherichia coli* expressing FimH, a bacterial lectin specific for high mannose, to vaginal epithelia [58, 59]. High mannose epitopes found on the gp120 coat protein of HIV-1 bind to endogenous lectins DC-SIGN and the macrophage mannose receptor (MMR) and aid in the infection of macrophages and dendritic cells [60, 61]. Binding of vaginal MMR by gp120 on HIV-1 may also play a role in sexual transmission of HIV-1 [62]. Native high mannose residues found on glycoproteins in vaginal fluids may thus act as natural inhibitors of these pathogenic interactions, helping to prevent viral entry and bacterial adhesion in women with normal microflora. The alteration in high mannose levels associated with abnormal microflora may alter the innate immune properties of the vaginal mucosa enabling enhanced pathogenesis in concordance with the increased infection risk.

### Exogenous Hormones Influence the Glycome

**Fig 4:**
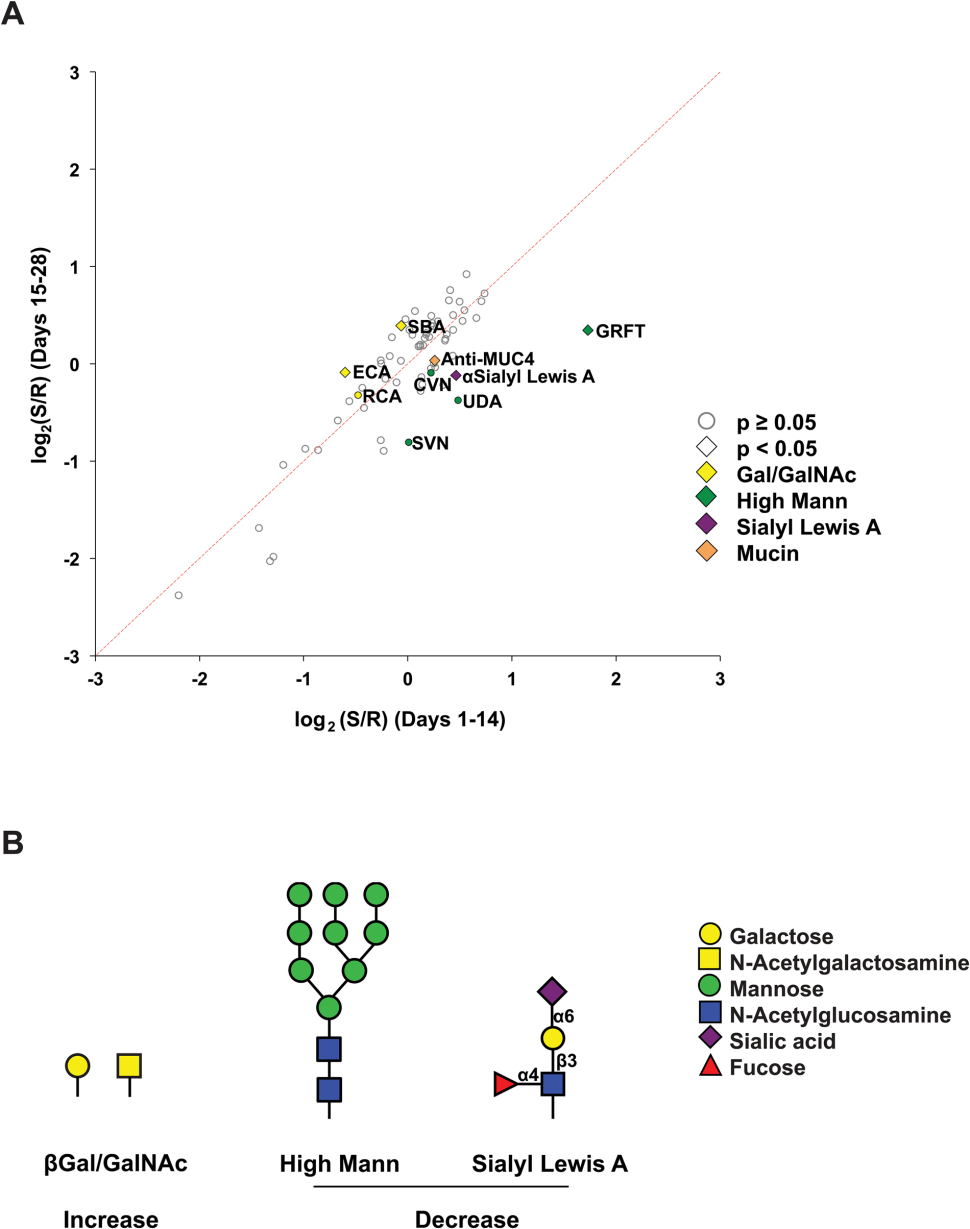
Effects of menstrual cycle on glycosylation of CVL. (A) Bi-plot of lectin microarray data for CVL from women in days 1–14 (x-axis) versus days 15–28 (y-axis). Graph shows average data for each lectin. Lectins and antibodies showing significant differences (p < 0.05) between the two groups are labeled with diamonds. Lectins with similar binding glycans are labeled in the same color (yellow: Gal/GalNAc; green: high mannose; purple: sialyl Lewis A). Lectins above the red dashed line showed increased expression levels during days 15–28 of the menstrual cycle compared to days 1–14. (B) Visual representation of glycans showing significant differences between the two groups.

**Fig 5:**
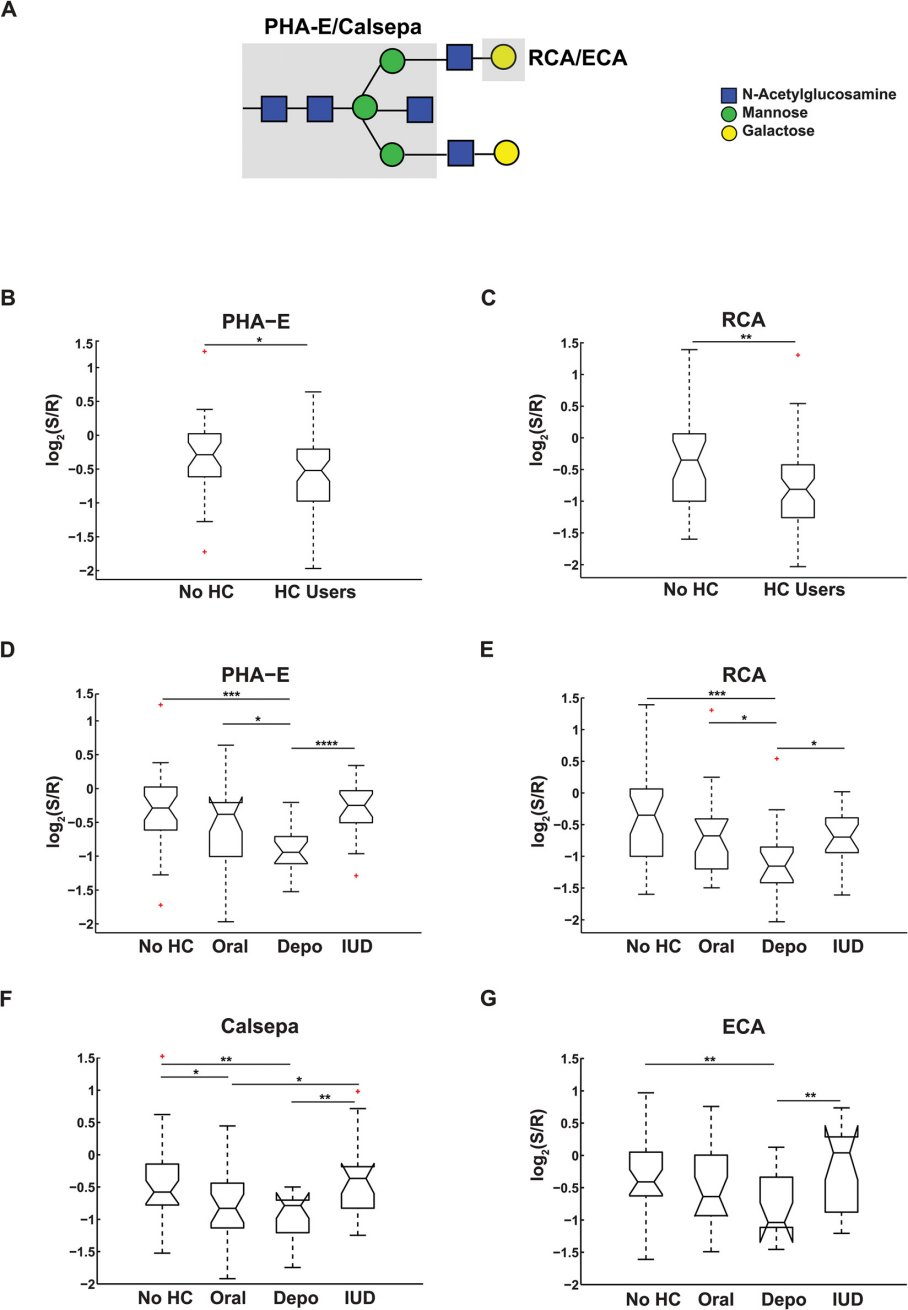
Effects of exogenous hormones on CVL glycome. (A) Representative *N*-linked complex glycan with bisecting GlcNAc. Lectin binding epitopes are shaded in grey. (B-C) Notched boxplot representation of binding levels of (B) PHA-E and (C) RCA for women on no hormonal contraceptives (No HC) or on hormonal contraceptives (HC). (D-G) Notched boxplot representation of detailed analysis of binding of (D) PHA-E, (E) RCA, (F) Calsepa and (G) ECA for women on oral contraceptives (Oral), Depo-Provera (Depo) or IUD in comparison to the No HC cohort. Significance: *, 0.01< *p* ≤0.05; **, 0.001< p ≤0.01; ***, 0.0001<p≤0.001; ****, p≤0.0001. Outliers are marked in red.

We reanalyzed our lectin microarray data for hormonal effects, examining the data for the 90 women with normal microflora. We excluded postmenopausal women, as we could not evaluate the status of their microbiome. We examined the effects of normal hormonal status on the CVL glycome by comparing women in days 1–14 of their menstrual cycle to women in days 15–28. We observed several changes associated with the menstrual cycle including a loss of GRFT binding in the later stages (p = 0.036, Fig 4), which is consistent with analysis in the companion paper by Moncla et al. To examine the effects of exogenous hormone contraceptives on glycomic composition of the CVL, we combined women in menstrual cycle without contraceptives (No HC, n = 31, Fig 5) and compared them to contraceptive users (HC Users, n = 59). Only two lectins gave statistically significant differences in signal for the cohorts. PHA-E, which binds to *N*-linked glycans containing bisecting GlcNAc [63], and RCA, which binds terminal galactose, both showed decreased binding to the CVL of contraceptive users (Fig 5A–5C, *p* = 0.046 and *p* = 0.002 respectively). We next ran a more detailed analysis, looking at the contribution of individual contraceptive cohorts (Oral, Depo-Provera and IUD) to the differences observed. All three contraceptive methods contain different synthetic hormones; combined oral contraceptives have estrogen and progesterone, Depo-Provera contains depo-medroxyprogesterone acetate, a progesterone derivative, and the IUD contains a different progesterone derivative, levonorgestrel. Depo-Provera users showed significantly lower levels of bisecting GlcNAc (PHA-E, Calsepa [64], Fig 5D and 5F, *p* = 0.0004 and *p* = 0.007 respectively) and terminal galactose (RCA, ECA, Fig 5E and 5G, *p* = 0.0009 and *p* = 0.004, respectively) than other contraceptive users and were the major contributor to the pattern observed for contraceptive use. In recent studies bisecting GlcNAc epitopes were found to enhance binding of IgG Fc to the FcγIIIa receptor leading to antibody-dependent cellular cytotoxicity [65]. Decrease in this epitope would presumably lower the levels of antibody-dependent immune response. This is consistent with recent work showing an anti-inflammatory effect by Depo-Provera on endocervical cells [66]. Oral contraceptive users also showed some glycomic changes in comparison to non-users, although the effects were less significant (0.01 < *p* < 0.05) and a clear glycomic pattern did not emerge. IUD users showed no real changes compared to the no-HC group. Our data suggests that both hormonal contraceptive composition and delivery method impact the vaginal glycome in ways that may affect immunity.

## Conclusions

The glycome of fluids from the cervico-vaginal tract plays an important role in the innate immune system, preventing pathogenic interactions and modulating immune activation. The study described herein and the accompanying work by Moncla et al (PONE-D-15-01714), which use cervical vaginal lavage samples (CVL) to represent the fluid and mucus from the lower reproductive tract, are the first to examine the role of hormonal contraceptives and microflora on glycosylation in this critical immunological fluid. Our data demonstrates that pathogenic microflora, such as that observed in bacterial vaginosis, have a profound effect on the CVL glycome overriding hormonal effects. These alterations may lower the immunological function of the cervico-vaginal fluids, enhancing the ability of secondary pathogens, such as HIV-1, to infect the host. Hormonal contraceptives also alter this glycome, with currently unknown implications. Both the route of contraceptive administration and the type of synthetic hormone used may play a role in these changes. These differences point out the need for further paired-patient studies examining the direct effects of different hormonal contraception agents on the glycome and on the innate immune protection provided by cervico-vaginal fluids.

## Supporting Information

S1 Data Processed Lectin Microarray Dataset used in analysis. Raw data is available at www.synapse.org (doi:10.7303/syn3243404).
(XLSX)

S1 Fig Lectin Microarray Comparison of Dialyzed and Undialyzed CVL. Lectin microarray analysis of matched dialyzed and undialyzed Cy3-labeled samples. The fluorescence was inhibited by free sugar in the CVL. Sample images are shown.
(TIFF)

S2 Fig Effect of hormones on glycans that change with BV. (A-H) Notched boxplot representation of binding levels of (A) SNA, (B) TJA-I, (C) ECA, (D) RCA, (E) AIA, (F) MNA-G, (G) GRFT and (H) SVN for women on no hormonal contraceptives (days 1–14 and 15–28) and on hormonal contraceptives (oral contraceptives (Oral), Depo-Provera (Depo) or IUD). Significance: *, 0.01< *p* ≤0.05; **, 0.001< p ≤0.01; ***, 0.0001<p≤0.001; ****, p≤0.0001. Outliers are marked in red. For all plots, significance levels between groups indicated by lines are as follows: *, 0.01< *p* ≤0.05; **, 0.001< p ≤0.01; ***, 0.0001<p≤0.001; ****, p≤0.0001. Outliers are marked in red. Differences due to hormone levels do not show the same general glycan patterns as BV vs. non-BV.
(TIF)

S3 Fig Effects of vaginal microflora on select glycans in women during days 1–14 of the menstrual cycle. (A-H) Notched boxplot representation of binding levels of (A) SNA, (B) TJA-I, (C) ECA, (D) RCA, (E) AIA, (F) MNA-G, (G) GRFT and (H) SVN for women at days 1–14 of the menstrual cycle with different flora states. The same effects caused by microflora were observed in these women, matching the trends seen in combined cohorts. Significance: *, 0.01< *p* ≤0.05; **, 0.001< p ≤0.01; ***, 0.0001<p≤0.001; ****, p≤0.0001. Outliers are marked in red.
(TIF)

S4 Fig Effects of vaginal microflora on select glycans in women on Depo-Provera. (A-H) Notched boxplot representation of binding levels of (A) SNA, (B) TJA-I, (C) ECA, (D) RCA, (E) AIA, (F) MNA-G, (G) GRFT and (H) SVN for women on Depo with different flora states. The same effects caused by microflora were observed in these women, matching the trends seen in combined cohorts. Significance: *, 0.01< *p* ≤0.05; **, 0.001< p ≤0.01; ***, 0.0001<p≤0.001; ****, p≤0.0001. Outliers are marked in red.
(TIF)

S5 Fig Effect of flora on levels of α2, 3-sialic acid as probed by MAL-I binding. Notched boxplot representation of binding levels of MAL-I to normal, intermediate and BV samples is shown. Outliers are marked red. The observed difference is not statistically significant (*p* = 0.4).
(TIFF)

S6 Fig Effect of BV on HPA binding. Notched boxplot representation of binding levels for HPA, a lectin that binds α-GalNAc, to normal, intermediate and BV samples is shown. None of the differences were statistically significant. Outliers are marked red.
(TIF)

S7 Fig Effect of BV on Lectins Binding Man_5_-Man_6_. Notched boxplot representation of binding levels for lectins that bind Man_5_-Man_6_ to normal, intermediate and BV samples are shown. (A) AMA, (B) ASA, (C) GNA, (D) NPA and (E) ConA. None of the differences were statistically significant. Outliers are marked red.
(TIF)

S1 Table Lectin Microarray Panel, Lectin Specificities and Print Conditions. (DOCX)
