# Supplementary figures and images for: Studying the Effects of Reproductive Hormones and Bacterial Vaginosis on the Glycome of Lavage Samples from the Cervicovaginal Cavity
Source: PLoS One. 2015 May 20;10(5):e0127021. doi: 10.1371/journal.pone.0127021 (PMC4439148; doi:10.1371/journal.pone.0127021)

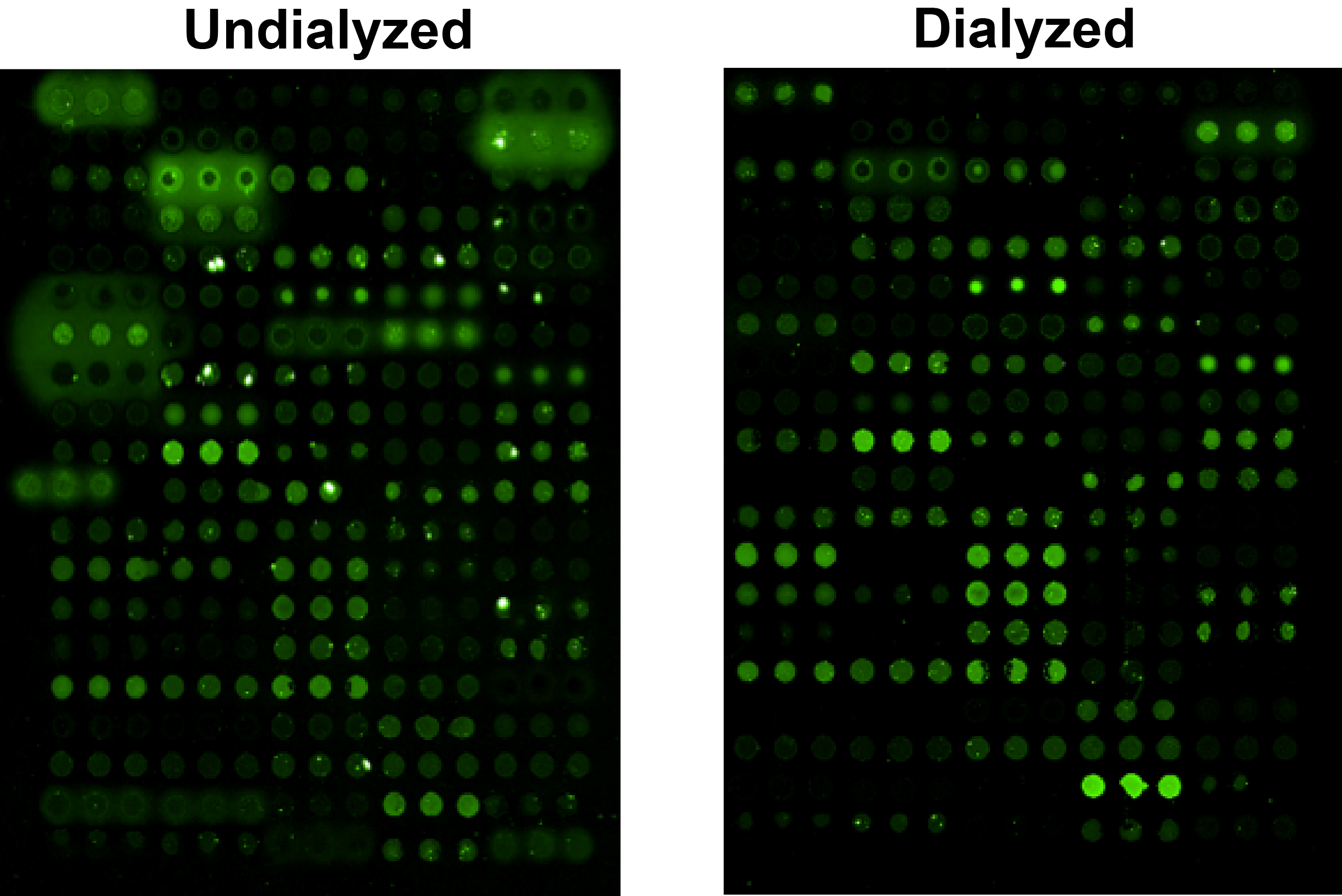

Supplement: S1 Fig — Lectin microarray analysis of matched dialyzed and undialyzed Cy3-labeled samples. The fluorescence was inhibited by free sugar in the CVL. Sample images are shown. (TIFF) [file pone.0127021.s002.tiff]

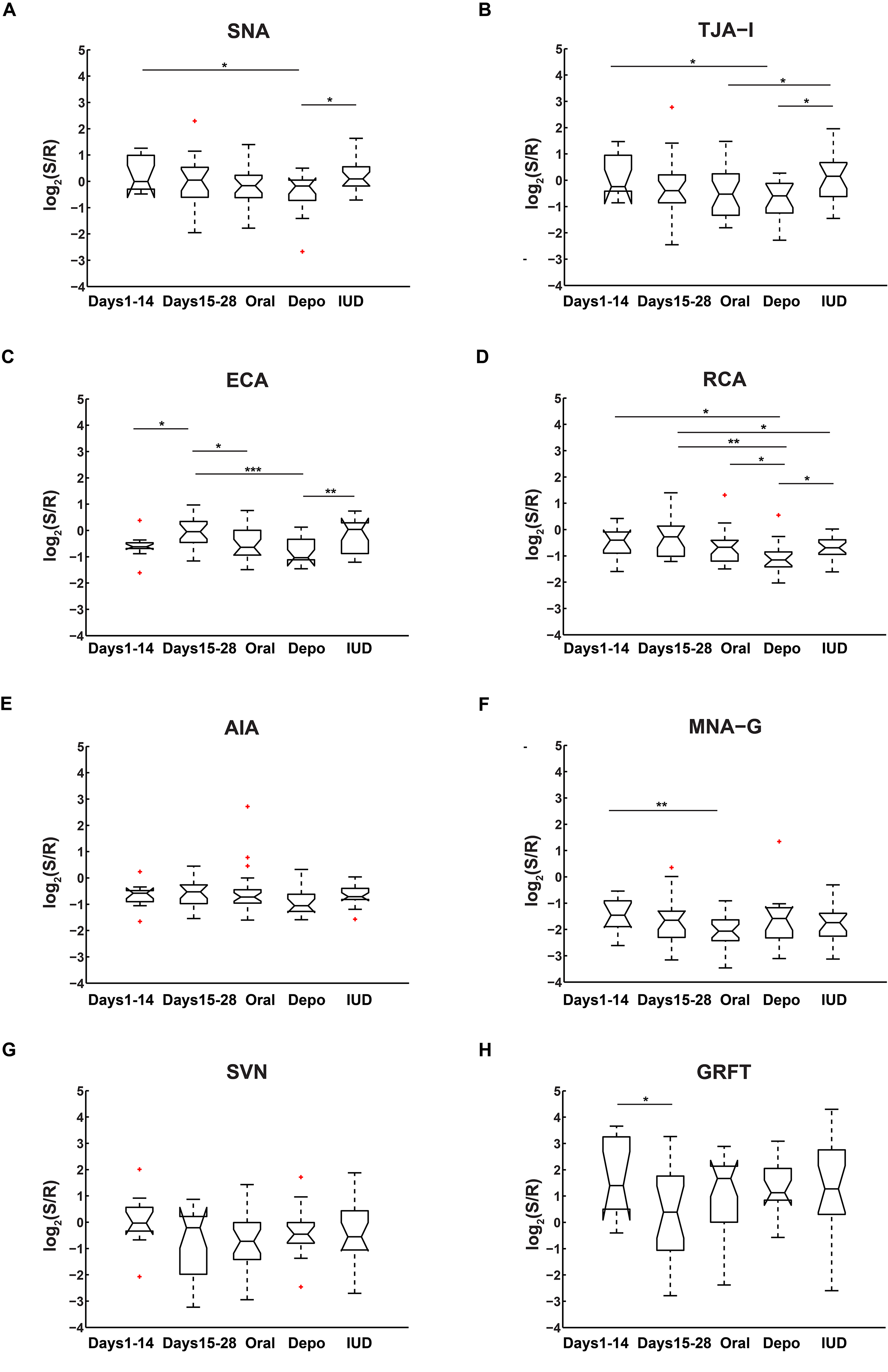

Supplement: S2 Fig — (A-H) Notched boxplot representation of binding levels of (A) SNA, (B) TJA-I, (C) ECA, (D) RCA, (E) AIA, (F) MNA-G, (G) GRFT and (H) SVN for women on no hormonal contraceptives (days 1–14 and 15–28) and on hormonal contraceptives (oral contraceptives (Oral), Depo-Provera (Depo) or IUD). Significance: *, 0.01< p ≤0.05; **, 0.001< p ≤0.01; ***, 0.0001<p≤0.001; ****, p≤0.0001. Outliers are marked in red. For all plots, significance levels between groups indicated by lines are as follows: *, 0.01< p ≤0.05; **, 0.001< p ≤0.01; ***, 0.0001<p≤0.001; ****, p≤0.0001. Outliers are marked in red. Differences due to hormone levels do not show the same general glycan patterns as BV vs. non-BV. (TIF) [file pone.0127021.s003.tif]

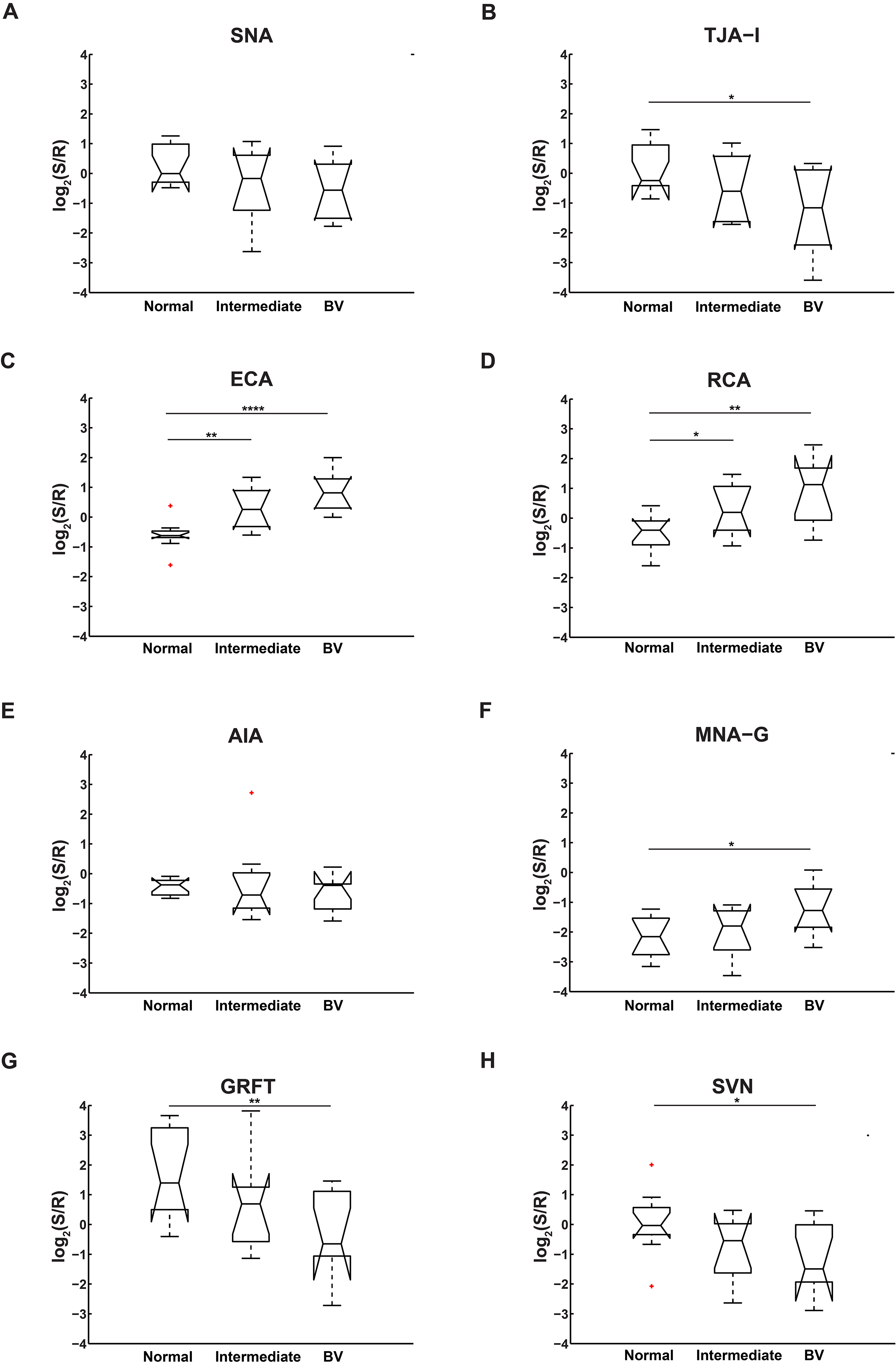

Supplement: S3 Fig — (A-H) Notched boxplot representation of binding levels of (A) SNA, (B) TJA-I, (C) ECA, (D) RCA, (E) AIA, (F) MNA-G, (G) GRFT and (H) SVN for women at days 1–14 of the menstrual cycle with different flora states. The same effects caused by microflora were observed in these women, matching the trends seen in combined cohorts. Significance: *, 0.01< p ≤0.05; **, 0.001< p ≤0.01; ***, 0.0001<p≤0.001; ****, p≤0.0001. Outliers are marked in red. (TIF) [file pone.0127021.s004.tif]

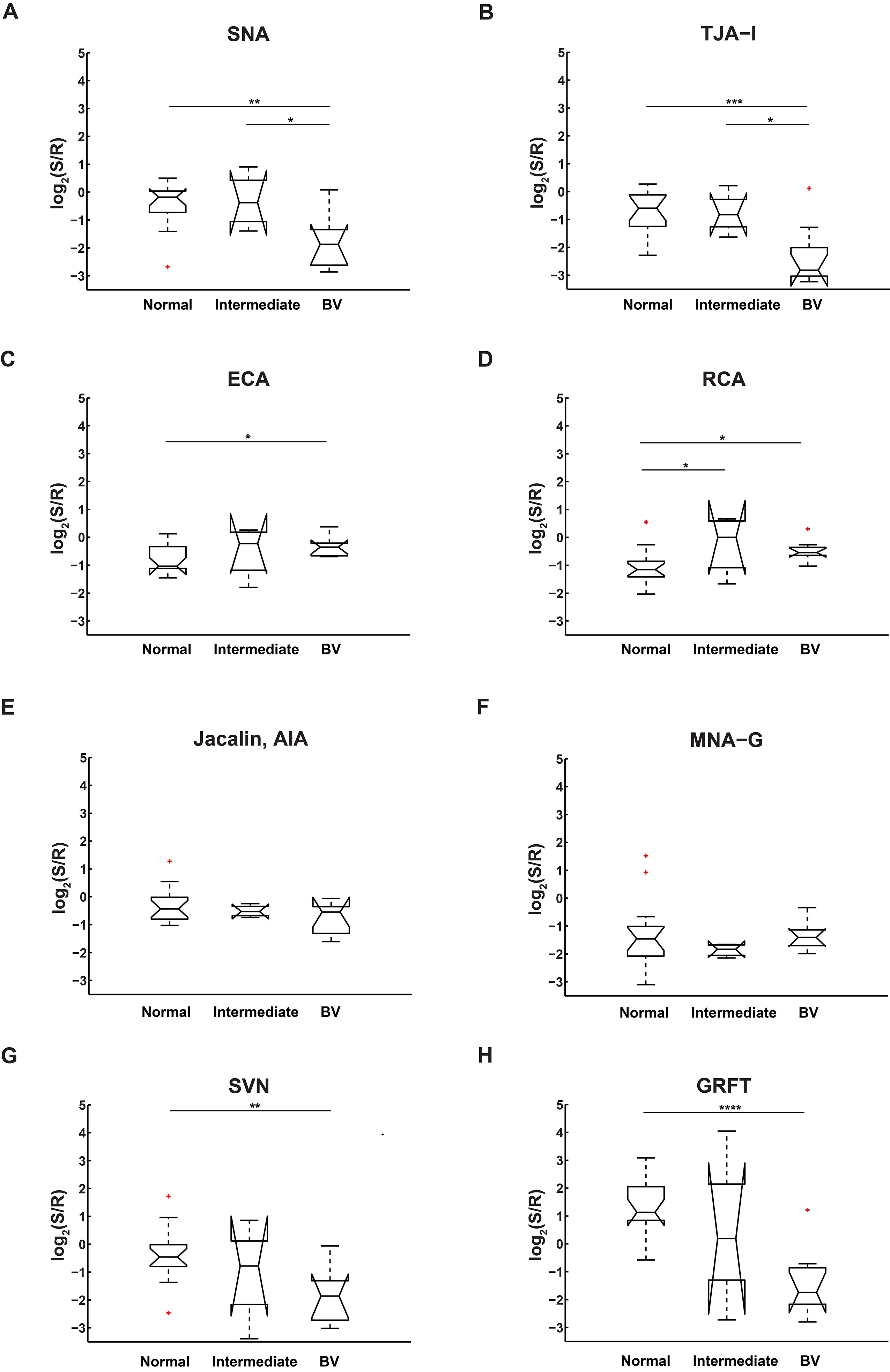

Supplement: S4 Fig — (A-H) Notched boxplot representation of binding levels of (A) SNA, (B) TJA-I, (C) ECA, (D) RCA, (E) AIA, (F) MNA-G, (G) GRFT and (H) SVN for women on Depo with different flora states. The same effects caused by microflora were observed in these women, matching the trends seen in combined cohorts. Significance: *, 0.01< p ≤0.05; **, 0.001< p ≤0.01; ***, 0.0001<p≤0.001; ****, p≤0.0001. Outliers are marked in red. (TIF) [file pone.0127021.s005.tif]

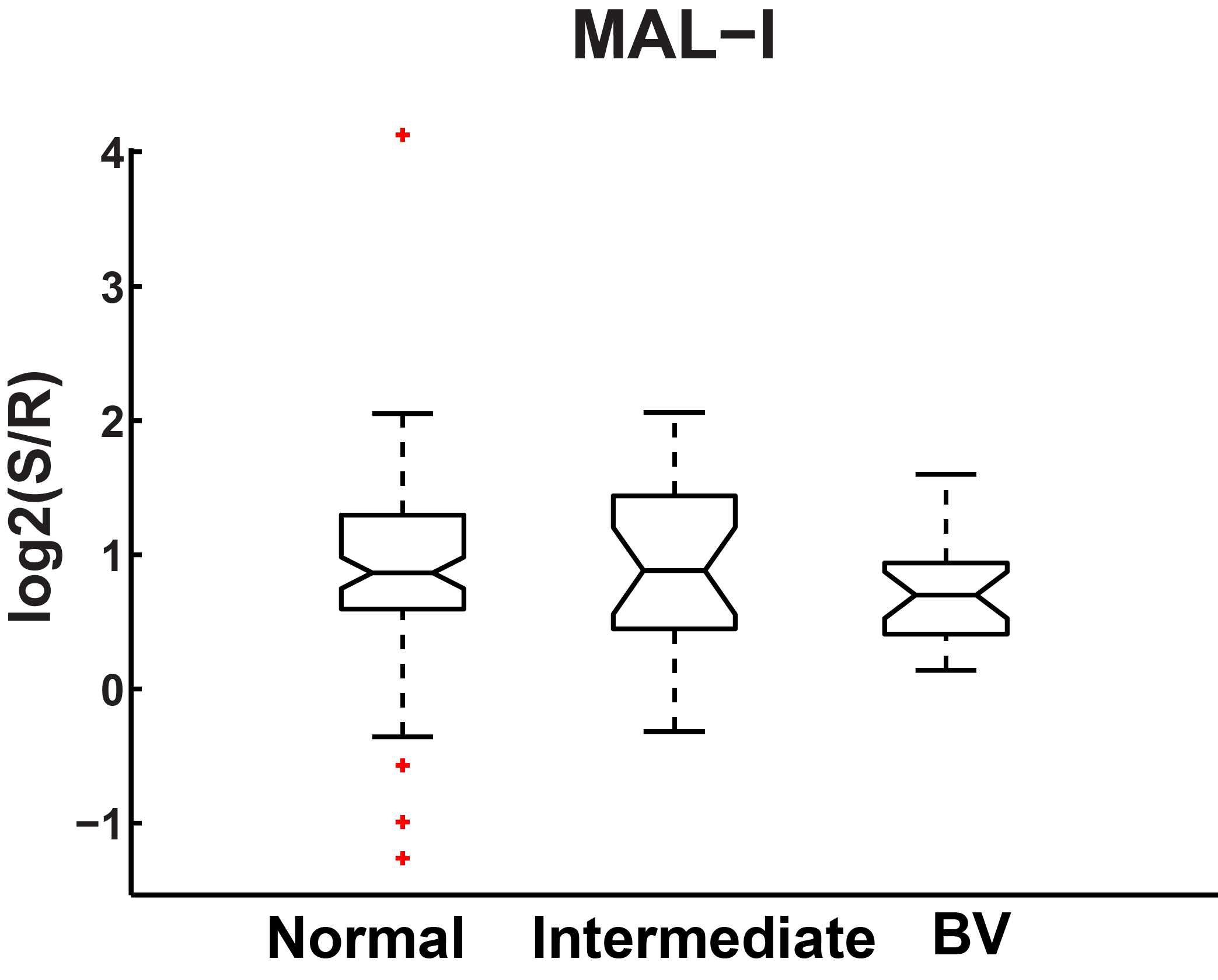

Supplement: S5 Fig — Notched boxplot representation of binding levels of MAL-I to normal, intermediate and BV samples is shown. Outliers are marked red. The observed difference is not statistically significant (p = 0.4). (TIFF) [file pone.0127021.s006.tiff]

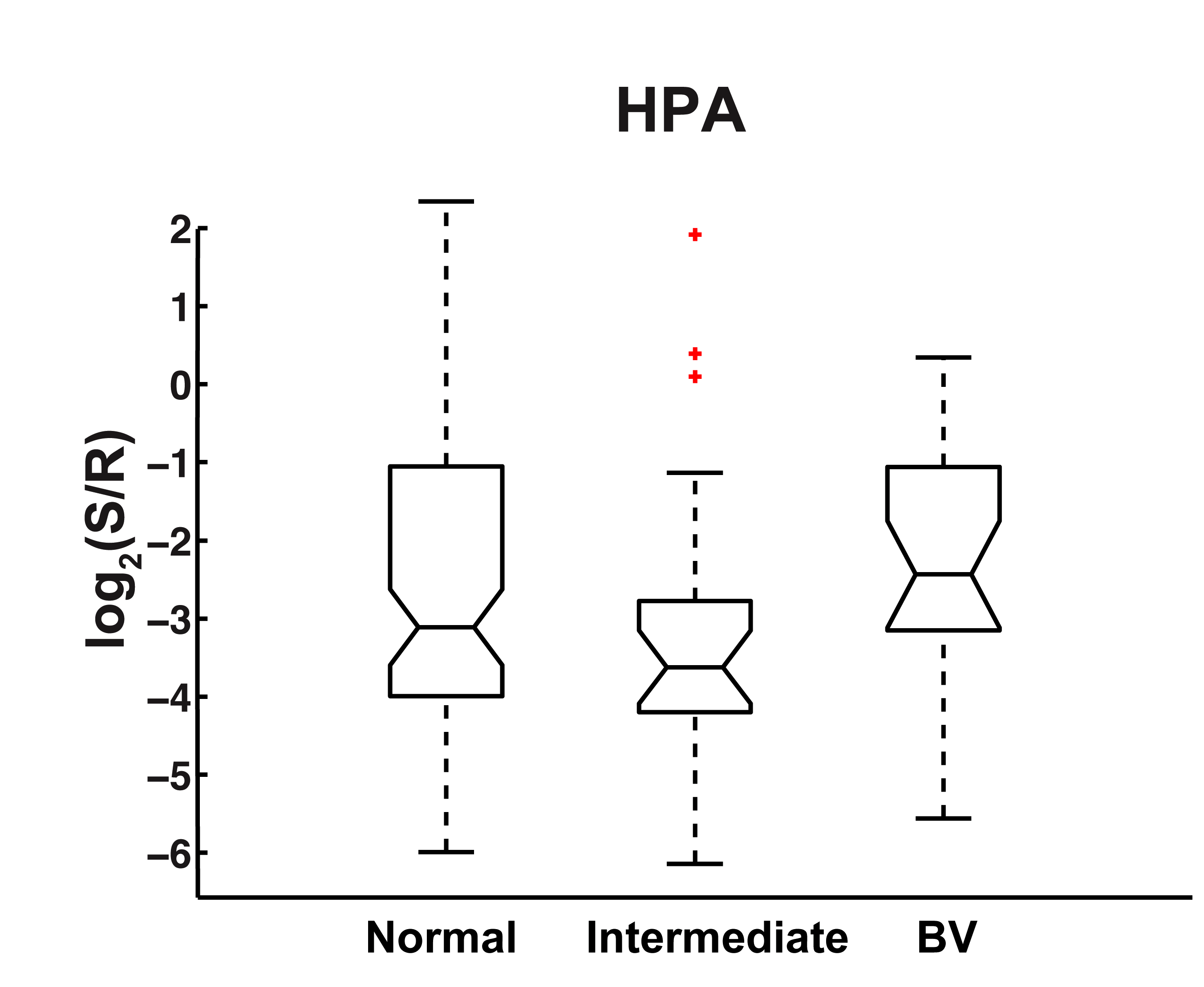

Supplement: S6 Fig — Notched boxplot representation of binding levels for HPA, a lectin that binds α-GalNAc, to normal, intermediate and BV samples is shown. None of the differences were statistically significant. Outliers are marked red. (TIF) [file pone.0127021.s007.tif]

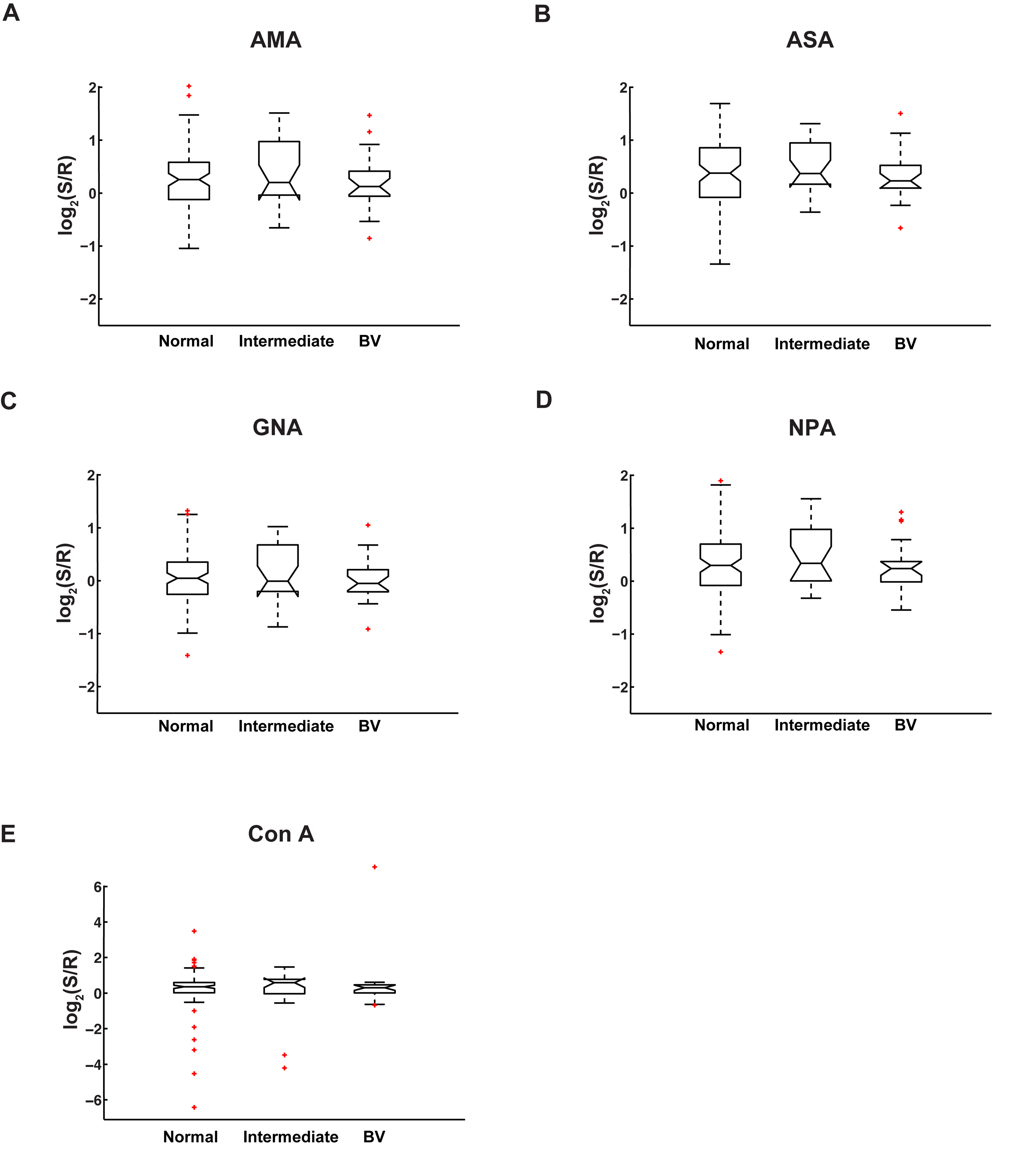

Supplement: S7 Fig — Notched boxplot representation of binding levels for lectins that bind Man5-Man6 to normal, intermediate and BV samples are shown. (A) AMA, (B) ASA, (C) GNA, (D) NPA and (E) ConA. None of the differences were statistically significant. Outliers are marked red. (TIF) [file pone.0127021.s008.tif]
