# Supplementary material for: Studying the Effects of Reproductive Hormones and Bacterial Vaginosis on the Glycome of Lavage Samples from the Cervicovaginal Cavity
Source: PLoS One. 2015 May 20;10(5):e0127021. doi: 10.1371/journal.pone.0127021 (PMC4439148; doi:10.1371/journal.pone.0127021)
Supplement: S1 Table — (DOCX) [file pone.0127021.s009.docx]

**Supporting Table 1**. Lectin microarray panel, lectin specificities and print conditions.

| **Abbreviation** | **Lectin source** | **Vendor** | **Print conc. µg/ml** | **Inhibitory monosaccharide** | **Rough specificity** |
| --- | --- | --- | --- | --- | --- |
| AAA | *Anguilla anguilla* | EY Laboratories | 1000 | Fuc | α-Fuc |
| AAL | *Aleuria aurantia* | Vector Labs | 500 | Fuc | Fuc |
| AOL | *Aspergillus oryzae* | TCI | 500 | Fuc | Fucα1-6 (core fucosylation),Fucα1,2Gal |
| UEA-I | *Ulex europaaeus I* | Vector Labs | 500 | Fuc | Fucα1-2Galβ1-4GlcNAc |
| PTLII | *Psophocarpus tetragonolobus* | Vector Labs | 1000 | Gal | β-GalNAc, Type II Blood H |
| TJA-II | *Trichosanthes japonica* | TCI | 500 | Lac | Fuca1-2Galb1-3/4GlcNAc, GalNAcβ1-4Galβ1 |
| PSA | *Pisum sativum* | Vector Labs | 500 | Man | Man |
| CCA* | *Cancer antennarius* | EY Laboratories | 1000 | Lac | 9-O-Acetyl NeuAc and 4-O-AcetylNeuAc |
| LFA | *Limax flavus* | EY Laboratories | 500 | Lac | α -NeuAc (O-glycans) |
| LPA | *Limulus polphemus* | EY Laboratories | 500 | Lac | α -NeuAc |
| MAL-I | *Maackia amurensis -I* | Vector Labs | 2000 | Lac | NeuAcα2-3LacNAc |
| MAL-II* | *Maackia amurensis -II* | Vector Labs | 1000 | Lac | NeuAcα2-3LacNAc |
| PSL | *Polyporus Squamosus* | EY Laboratories | 500 | Lac | NeuAcα-2-6LacNAc |
| SNA | *Sambucus nigra* | EY Laboratories | 500 | Lac | NeuAcα-2-6, (Lac core) |
| TJA-I | *Trichosanthes japonica* | TCI | 1000 | Lac | NeuAca2-6LacNAc or 6-SulfoLacNAc |
| PHA-L* | *Phaseolus vulgaris-L* | Vector Labs | 500 | Gal | Complex triantennary N-linked glycans |
| ECA | *Erythrina cristagalli* | Vector Labs | 500 | Gal | GalNAcβ1-4GlcNAc, Galβ1-4GlcNAc |
| RCA | *Ricinus communis agglutinin B* | Vector Labs | 1000 | Lac | Terminal β-Gal, terminal LacNAc |
| PHA-E | *Phaseolus vulgaris-E* | Vector Labs | 500 | Lac | Complex N-linked (bisecting GlcNAc) |
| CA* | *Colchicum autumnale* | EY Laboratories | 500 | Gal | Terminal β-Gal, α- and β-GalNac |
| BPA | *Bauhinia purpurea* | Vector Labs | 500 | Gal | Galβ1-3 or GalNAcβ1-4 more weakly |
| APA | *Abrus precatorius* | EY Laboratories | 500 | Gal | Gal β-1,3GalNAc (TF antigen) > Gal |
| GS-I* | *Griffonia simplicifolia I* | Vector Labs | 1000 | Gal | α-Gal, some GalNAc |
| APP | *Aegopodium podagraria* | EY Laboratories | 500 | GalNAc | GalNAc>Lacose>Galactose |
| BDA | *Bryonia dioica* | EY Laboratories | 500 | Gal | GalNAc |
| Blackbean | *Black bean* | EY Laboratories | 2000 | Lac | GalNAc |
| CAA | *Caragana arborescens* | EY Laboratories | 500 | Gal | GalNAc/Gal (monosaccharides best) |
| CSA | *Cystisus scoparius* | EY Laboratories | 1000 | Gal | β-GalNAc, terminal |
| IRA* | *Iris Hybrid* | EY Laboratories | 1000 | Gal | GalNAc(GalNAc α-1,3)Gal>GalNAc>Gal |
| VVA* | *Vicia villosa* | Vector Labs | 500 | Gal | α-Linked terminal GalNAc, GalNAcα-1,3 Gal |
| WFA* | *Wisteria floribunda* | Vector Labs | 500 | Gal | GalNAc |
| ASA | *Allium sativum* | EY Laboratories | 1000 | Man | Mannose |
| AMA | *Allium moly* | EY Laboratories | 1000 | Man | D-Mannose |
| Calsepa | *Calystegia sepium* | EY Laboratories | 500 | Man | Man/maltose |
| Con A | *Canavalia ensiformis* | EY Laboratories | 1000 | Man | Branched and terminal mannose [High-Man, Manα-1,6 (Manα-1,3)Man] |
| CVN | *Cyanovirin* | Barry O'Keefe's laboratory (National Cancer Institute) | 1000 | Man | α-1,2 Mannose |
| GNA | *Galanthus nivalis* | EY Laboratories | 1000 | Man | Terminal α-1,3mannose |
| GRFT | *Griffithsin* | Barry O'Keefe's laboratory (National Cancer Institute) | 1000 | Man | Mannose,GlcNAc |
| HHL* | *Hippeastrum Hybrid* | Vector Labs | 1000 | Man | α-1,3 Mannose and α-1,6 mannose |
| NPA | *Narcissus pseudonarcissus* | EY Laboratories | 1000 | Man | Terminal and internal Man |
| SVN | *Scytovirin* | Barry O'Keefe's laboratory (National Cancer Institute) | 500 | Man | α-1,2 Mannose |
| TL | *Tulipa sp.* | EY Laboratories | 1000 | GlcNAc | Man3 core, bi- and tri-antennary complex-type N-glycan, GalNAc |
| UDA | *Uritica dioica* | EY Laboratories | 1000 | GlcNAc | GlcNAc β-1,4 GlcNAc oligomers and high mannose epitopes |
| VVA (mann)* | *Vicia villosa* | EY Laboratories | 500 | Gal | Man |
| VFA | *Vicia faba* | EY Laboratories | 500 | Gal | Man>Glc>GlcNAC |
| LcH | *Lens culinaris* | Vector Labs | 500 | Man | Complex |
| Jacalin, AIA | *Artocarpus integrifolia* | Vector Labs | 500 | Gal | O-glycosidically linked oligosaccharides, preferring Gal (β-1,3)GalNAc |
| EEL | *Eunonymus europaeus* | Vector Labs | 1000 | Lac | Blood group B antigen, Gal α-1,3 Gal |
| MPL* | *Maclura pomifera* | Vector Labs | 1000 | Gal | Gal β-1,3 GalNAc, GalNAc |
| PNA* | *Arachis hyogaea* | Vector Labs | 2000 | Gal | Terminal Gal β-OR |
| VGA* | *Vicia graminea* | EY Laboratories | 1000 | Gal | O-linked Gal β-1,3 GalNAc |
| VRA | *Vigna radiata* | EY Laboratories | 1000 | Gal | α-Galactose |
| ACA | *Amaranthus Caudatus* | EY Laboratories | 1000 | Lac | Gal β-1,3 GalNAc (the T antigen) |
| DBA | *Dolichos biflorus* | Vector Labs | 1000 | Gal | GalNAc α-OR (GalNAcα-1,3 GalNAc) and Blood group A antigen, |
| SBA | *Glycine max* | Vector Labs | 500 | Gal | α- or β-Linked terminal GalNAc, GalNAc α-1,3 Gal |
| SJA | *Sophora japonica* | Vector Labs | 500 | Gal | GalNAc |
| HAA | *Homarus Americanus* | EY Laboratories | 1000 | Gal | Terminal GalNAc |
| HPA | *Helix pomatia* | EY Laboratories | 1000 | Gal | α -Linked terminal GalNAc |
| SNA-II* | *Sambucus nigra* | EY Laboratories | 500 | Gal | GalNAc linked alpha to C-2, C-3 or C-6 hydroxyl gropup of galactose |
| PTLI* | *Psophocarpus tetragonolobus* | Vector Labs | 1000 | Gal | α-GalNAc, A-antigen |
| PTA | *Psophocarpus tetragonolobus* | EY Laboratories | 500 | Gal | Gal |
| LBA | *Phaseolus lunatus* | EY Laboratories | 1000 | Gal | GalNAc α-1,3 [Fuc α-1,2]Gal |
| DSA* | *Datura stramonium* | Vector Labs | 500 | Lac | GlcNAc β-1,4 GlcNAc oligomers and LacNAc (Gal β 1-4 GlcNAc) |
| GS-II | *Griffonia simplicifoia* | Vector Labs | 500 | GlcNAc | terminal GlcNAc |
| UEA-II | *Ulex europaaeus II* | EY Laboratories | 1000 | GlcNAc | Oligomers of β-1,4 GlcNAc |
| CPA | *Cicer arietinum* | EY Laboratories | 1000 | Lac | Complex |
| RPA | *Robinia pseudoacacia* | EY Laboratories | 500 | GlcNAc | Complex |
| LEA | *Lycopersicon esculentum* | Vector Labs | 500 | GlcNAc | β-1,4GlcNAc oligomers |
| STA* | *Solanus tuberosum* | Vector Labs | 500 | GlcNAc | GlcNAc oligomers, LacNAc |
| WGA | *Triticum vulgare* | Vector Labs | 1000 | GlcNAc | β-GlcNAc, sialic acid, GalNAc |
| Cholera Toxin | *Cholera Toxin from Vibrio cholerae* | EY Laboratories | 2000 | Lac | glycolipid |
| MOA | *Marasmium oreades* | EY Laboratories | 500 | Gal | Gal α-1,3 Gal and Gal α-1,3 Gal β-1,4GlcNAc |
| MNA-G | *Morniga sp.* | EY Laboratories | 1000 | Gal | GalNAc α, Tn antigen |
| Lotus* | *Lotus tetragonolobus* | Vector Labs | 500 | Fuc | Terminal α-Fuc, Lewis x |
| IAA | *Iberis amara* | EY Laboratories | 500 | GalNAc | GalNAc |
| GS-I | *Griffonia simplicifolia I* | EY Laboratories | 1000 | Gal | α-Gal, some GalNAc |
| GafD^†^ | *Escherichia coli F17 fimbriae* | Recombinant | 500 | GlcNAc | β-GlcNAc |
| Galectin-I^†^* | *Homo Sapiens* | Recombinant | 500 | Gal | Galβ1-4GlcNAc |
| PA-IL^†^ | *Pseudomonas Aeruginosa* | Recombinant | 500 | Gal | Terminal α-Gal |
| PA-II S22A^†^ | *Pseudomonas Aeruginosa* | Recombinant | 500 | Man | Mannose |
| RS-IIL A22S^†^ | *Ralstonia solanacearum Nonfimbriae* | Recombinant | 500 | Fuc | Fuc |
| PNGase F D60N^†^* | Peptide -N-Glycosidase F | Recombinant | 500 | Man | N-linked glycans |
| Anti-DCSIGN* |  | Abcam | 100 |  | DC-SIGN |
| Anti-HSA |  | Abcam | 100 |  | HSA |
| Anti-MUC1 |  | Abcam | 100 |  | MUC1 |
| Anti-MUC4 |  | Abcam | 100 |  | MUC4 |
| Anti-MUC5AC |  | Abcam | 100 |  | MUC5AC |
| Anti-MUC6* |  | Sdix | 100 |  | MUC6 |
| Anti-MUC7 |  | Novus Biologicals | 100 |  | MUC7 |
| 7LE |  | Santa Cruz Biotechnology | 100 |  | Lewis a |
| SPM110 |  | Santa Cruz Biotechnology | 100 |  | Sialyl Lewis a |
| 2-25LE |  | Santa Cruz Biotechnology | 100 |  | Lewis b |

Print Buffer: 0.005% Tween in PBS pH 7.6

†Print Buffer for Oriented Lectins: 100 mM Glutathione in 50 mM sodium borate pH 8.5

‡Abbreviations: Gal (Galactose), GlcNAc (N-acetyl-Dglucosamine), GalNAc (N-acetyl-D-galactosamine), Fuc (Fucose), Lac (Lactose), Man (Mannose), NeuAc (Sialic Acid).

* Lectins that did not pass quality control and were omitted from further analysis.
